# Testosterone Administration Related Differences in Brain Activation during the Ultimatum Game

**DOI:** 10.3389/fnins.2016.00066

**Published:** 2016-03-01

**Authors:** Eleni Kopsida, Jonathan Berrebi, Predrag Petrovic, Martin Ingvar

**Affiliations:** Department of Clinical Neuroscience, MR Research Center and Osher Center for Integrative Medicine, Karolinska InstitutetStockholm, Sweden

**Keywords:** testosterone, Ultimatum Game, gender, aggression, dlPFC

## Abstract

A plethora of studies on the Ultimatum Game have shown that responders forfeit the rule of profit maximization and punish unfair proposers, by rejecting their offers. This behavior has been linked to increased amygdala, insula, and dorsolateral prefrontal cortex activation. Studies have suggested a potential role of testosterone in the Ultimatum Game albeit with inconsistent findings. In the present study, we sought to further investigate the role of amygdala and testosterone in the Ultimatum Game, by conducting a double-blinded, single-administration study. Sixty milligram of Tostrex was administered to male and female healthy volunteers, 3 h prior to undergoing an fMRI session, during which they played a standard version of the Ultimatum Game. The behavioral analysis revealed a statistical trend, as participants in the testosterone group tended to accept a greater number of unfair offers than participants in the placebo group, irrespectively of gender. In terms of fMRI results, for the main contrast unfair>fair offers, the testosterone group displayed a greater activation in the right dlPFC compared to the placebo group. Increased testosterone levels were related to greater caudate activity. Our findings suggest a complex role of testosterone in social behavior and decision-making.

## Introduction

The previous standard definition on rational behavior posits that individuals act to maximize their profit when faced with an economic decision (Lee, 2008). However, later research on decision-making and neuroeconomics has shown that human beings frequently deviate from this pattern (Sanfey, 2007). A behavioral paradigm frequently used to investigate this process is the Ultimatum Game in which a proposer has a set amount of money that he/she splits with the receiver. The receiver can only accept or reject the offer that the proposer makes. In case of acceptance both parties receive the respective amount according to the proposed split, while in case of rejection both parties get nothing (Güth et al., 1982). Extensive data on the Ultimatum Game has revealed that, when confronted with an offer that they deem unfair, responders tend to reject that offer, even though this results in loss of money (Rilling and Sanfey, 2011). In other words, they act against the rule of profit maximization. This behavior has been attributed to either upholding a social norm of fairness by the act of punishment at their own expense or alternatively the explanation has been that the rejection is compatible with an impulsive response based on aggression and anger (Pillutla and Murnighan, 1996). Unfair offers induce more intense physiological reactions, indicating emotional arousal (Van't Wout et al., 2006) and are related to the activation of brain regions that are associated with negative affect, e.g., the anterior insula (Sanfey et al., 2003). Recently we demonstrated that the rejection behavior was also associated with an immediate amygdala activation (Gospic et al., 2011). Importantly, amygdala activation has previously been associated with unfair divisions (Haruno and Frith, 2010), framing and loss aversion (De Martino et al., 2006, 2010), hypothetical bias (Gospic et al., 2014), while lesions to this region have been related to aberrant behavior in the Ultimatum Game (Scheele et al., 2012).

Unfair offers potentially evoke a motivational conflict, as it entails arbitration between behaviors based on the immediate emotional reaction leading to rejection of the offer, and an act toward their self-interest to maximize their profit by accepting the proposal. This latter mechanism has been shown to be related to activity in the prefrontal areas, such as the anterior cingulate cortex (ACC) and the dorsolateral prefrontal cortex (dlPFC; Sanfey et al., 2003; Grecucci et al., 2013). It has been suggested that decision-making processes, including the Ultimatum Game, are guided by two interactive, yet separate, systems; one that relies on emotional processes and one that is based on deliberative processes (Sanfey et al., 2006). The emotional system entails automatic, low-level processes, and recruits mainly subcortical areas, such as the amygdala and the striatum, as well as the insula. The deliberation system is related to high-level cognitive processes and recruits mainly frontal cortical areas, such as the dlPFC (Sanfey et al., 2006).

In the present study we sought to further investigate the role of amygdala in the immediate response to unfair offers in the Ultimatum Game by manipulating the balance between these two systems. The manipulation consisted of a single exogenous dose of testosterone administered to healthy male and female volunteers. The reason for this was twofold. Firstly, testosterone has been related to increased amygdala activation during processing of emotional stimuli (Eisenegger et al., 2011). Secondly, a plethora of animal and human studies have yielded a role for testosterone in social interaction (McCall and Singer, 2012). High testosterone levels could promote both prosocial and anti-social behavior, depending on the context (Bos et al., 2012). On one hand, in a challenging environment, high testosterone levels have been related to high status seeking and dominance, to low levels of empathy and stress, signals denoting a bias toward competition and potential confrontation (Eisenegger et al., 2011). On the other hand, high levels of testosterone could promote prosocial behavior and parochial altruism (Everett et al., 2015). In the Ultimatum Game, the role of testosterone has been inconclusive. Specifically, testosterone has been shown to either have a null effect (administration studies of exogenous testosterone; Zethraeus et al., 2009; Eisenegger et al., 2010) or to increase rejection of unfair offers (correlational studies of endogenous testosterone levels) (Burnham, 2007; Mehta and Beer, 2010). This discrepancy could be attributed to different reasons. Both dosing and the form of administration could play a part, as well as the potential differential effects of endogenous and exogenous testosterone. Furthermore, gender may affect the response to testosterone. This is of importance as, apart from the study by Mehta and Beer (2010), where both genders were included (but with a moderate sample size of 32), the other aforementioned studies were conducted on one gender only (both administration studies included only women, while the study by Burnham included only men). Given the debate on hormonal influences on aggression and social interaction it is of interest to decipher the precise role of testosterone in the Ultimatum Game by including both genders under the same experimental protocol (Carre and Olmstead, 2015). Of note it is also the fact that some of our previous studies on decision-making have indicated gender differences, and this study was powered to test these. Briefly, in the Ultimatum Game male participants showed greater amygdala activation in relation to unfair offers compared to female participants (Gospic et al., 2011), while, in a recent study on hypothetical bias, they displayed a lower hypothetical bias (Gospic et al., 2014), implying gender differences in choice behavior.

Thus, healthy male and female participants received a single exogenous dose of testosterone, prior to engaging as responders in an Ultimatum Game. This was the first study, where testosterone manipulation was applied to both genders using the same protocol. An event-related fMRI design was implemented, which allowed for an accurate timing of the amygdala BOLD activation (Gospic et al., 2011). Our main hypothesis was that testosterone administration would either have an effect on responders' performance on the Ultimatum Game, by means of increasing the rejection rate of unfair offers. Given some prior contributions to the literature an alternative outcome could be a null effect. We also hypothesized that an increase of rejection rate would be related to an increased amygdala activity during unfair offers (Gospic et al., 2011).

## Methods

### Participants

Sixty-eight healthy volunteers (28 women; mean age = 23.4, s.d = 4.6) took part in a double-blinded, placebo-controlled, single-dose administration study (placebo group: *N* = 34; testosterone group: *N* = 34). None of the participants had any neurological or neuropsychiatric disorders nor were they taking any medication (apart from mild allergy medications). All participants were screened for MRI-contraindications, such as presence of metallic objects in the body, hearing implants etc. Furthermore, a clinical scan (FLAIR) was included in the fMRI session, which was subsequently examined by clinical radiologists. All women were on oral contraception (25 were on monophasic combined contraceptive pill, two were on progesterone-only pill, and one was on triphasic combined contraceptive pill). All had normal or corrected-to-normal visual acuity and normal color vision. Sixty-three were right-handed (five were left-handed). All participants spoke fluent Swedish. This study was carried out in accordance with the recommendations of the local governmental ethics committee (EPN) in Stockholm, Sweden. All subjects provided written informed consent in accordance with the Declaration of Helsinki.

### Procedure

All participants visited the lab twice. The first visit took place in the morning, while the second visit took place in the afternoon. During the first session, participants received information on the study and signed a consent form, filled in personality trait questionnaires (see Supplementary Material), and provided a blood sample, in order to ascertain their baseline testosterone levels. The second session took place approximately 2 weeks later (average interval = 18 days). In order to do the imaging study at the peak serum testosterone concentration (Eisenegger et al., 2013) all participants were asked to apply the gel 3 h prior to their arrival to the lab. Upon their arrival, participants provided another blood sample and filled in two questionnaires; STAI-STATE to measure anxiety and AQ-RSV to measure aggression (Forsberg and Björvell, 1993; Prochazka and Ågren, 2001).

Before the beginning of the fMRI session, the rules of the Ultimatum Game were explained. Participants were also asked whether they would like to play the role of the proposer in future studies, in an attempt to frame the experimental setting as more believable. They were also told that the proposers in the economic game were previous participants of the study. Participants were then asked to complete a questionnaire, in order to ascertain that they had fully understood the rules of the game. Furthermore, they were informed that upon completion of the game, three trials would be randomly picked and paid out in real money pending the results of those trials. During the fMRI session and prior to the Ultimatum Game, participants engaged in a passive monetary task (Wheel of Fortune; see Supplementary Material), in an attempt to further explore any effect of testosterone on reward-related processes.

After the completion of the fMRI session, participants viewed pictures of the proposers and were asked to rate their likeability on a VAS scale. They were also given two questionnaires to fill in. In the first questionnaire they had to rate the perceived unfairness of the offers that they had previously seen; in the second they had to indicate the group that they thought they belonged to (placebo or testosterone).

### Hormone preparations

The testosterone group received a single dose of testosterone gel (3 g), containing 60 mg testosterone (Tostrex, ProStrakan Ltd, U. K.). The placebo group received a placebo gel. Participants applied the gel to their inner thighs, after receiving oral and written instructions. Participants were asked to send a text to the experimenter upon application of the gel, so that the application time could be verified. All participants reported that they followed this instruction. No additional confirmation of application procedure was present (e.g., participants returning the empty gel kit for verification).

#### Hormone analysis

Serum concentration of testosterone was determined by isotope dilution and mass spectrometric detection. The measurement was performed with Ultra Performance HLPC (UPLC) and tandem mass spectrometry (LC-MS/MS) using positive electrospray. The areas under the chromatographic peaks of Testosterone (Transitions m/z 289–97 and m/z 289–109) were recorded (corresponding transitions of internal standards were m/z 292–97). The ratio between the transition surfaces were calculated and checked against the ratio of the calibrator. The ratio of the surfaces between the analyse and the internal standard were evaluated against the respective calibration curve, and the concentrations were calculated. For statistical comparisons, testosterone values were standardized within gender, using SPSS software (IBM, SPSS Statistics 21).

### Ultimatum game

**Figure 1:**
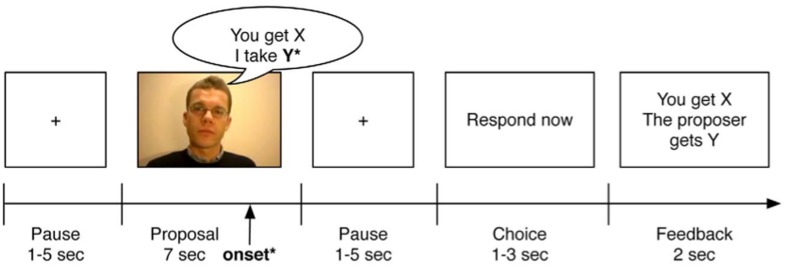
**Example of an experimental trial of the Ultimatum Game (picture taken with permission from Gospic et al., 2011)**. All participants watched 45 clips, each with a different proposer. The fMRI onset was set to the moment, when the proposer uttered the last word, thus denoting the fairness of the offer (fair or unfair).

All participants played a standard Ultimatum Game, assuming the role of the responder, with 45 different proposers (22 males, 23 females), while undergoing fMRI (Figure 1). The experimental design was identical to the design implemented previously in our lab (see Gospic et al., 2011 for more details). Briefly, all participants encountered 15 fair, 15 unfair, and 15 neutral proposals. According to the instructions, participants had to accept or reject the offer, by pressing the “yes” and the “no” button respectively. In the fair offers, the responder would get 50% and the proposer 50% of the money, while in the unfair offers the responder would get 20% and the proposer 80% of the money. The fair and unfair offers were designed to yield three different stake levels. Each participant encountered six different types of offers: six 50/50 offers, seven 20/80 offers, five 175/175 offers, five 50/200 offers, four 250/250 offers, and three 100/400 offers. In the neutral condition, participants watched videos of the proposers saying “this is not a proposal,” to which they had to respond “no.” For each participant, the videos of the proposers were randomly assigned, in order to control for a potential proposer by offer interaction. The average financial compensation per participant was 569 SEK.

### Data analysis

Data were analyzed using SPSS software (IBM, SPSS Statistics 21). Analysis was mainly conducted using non-parametric testing, as the data were either not normally distributed or were on a nominal (binary) scale. The analysis focused on the rejection of unfair offers. The effect of the treatment on rejection rate was analyzed using Mann–Whitney non-parametric test. To study a potential interaction with gender and offer stake, a probit model (GEE) was performed, with group, gender and offer stake as the predictors, and individual choices (accept/reject) as the dependent variable. Comparisons between groups on the questionnaire data were conducted using Mann–Whitney non-parametric test. In order to test for any effect of session (baseline, treatment) within the two groups (testosterone and placebo) Wilcoxon signed-rank test was implemented. To investigate potential gender differences on the AQ-RSV questionnaire after treatment, Mann–Whitney non-parametric test was conducted. The effect of treatment on the general likeability of the proposer was assessed with an independent *t*-test. To test for any testosterone belief effect a chi-square analysis was performed. Correlations, where appropriate, were performed using Spearman's r. Multiple comparisons, when appropriate, were performed using Bonferroni correction. Effect sizes are reported using Pearson's correlation coefficient r.

### Neuroimaging analyses

#### Data acquisition

Structural and functional data were acquired with a GE 3.0T scanner (Discovery MR750, GE) and a 32-channel coil (NOVA Medical). A high-resolution structural T1-weighed image (BRAVO sequence) was collected in 180 sagittal 1 × 1 × 1 mm thick slices, with *TR* = 7.9 ms, *TE* = 3.1 ms and flip angle = 12°. Before the beginning of the Ultimatum Game, a B0 map session (*TR* = 7.5 ms, *TE* = 6.8 ms (ΔTE = 2) flip angle:45°, field of view: 22 cm) was conducted, which was subsequently utilized in the analysis to correct for susceptibility artifacts (BO map sequence was acquired from http://rsl.stanford.edu/research/software.html). The Ultimatum Game was split into two fMRI sessions (23 and 22 trials, respectively). The T2^*^ weighted echoplanar image (EPI) sequence had the following protocol: number of slices = 45; slice thickness: 3 mm; *TR* = 2500 ms; *TE* = 30 ms; field of view: 22 cm, matrix size: 72 × 72. Slices were acquired in an interleaved order.

#### Data pre-processing and analysis

The fMRI data were analyzed with SPM 12 (http://www.fil.ion.ucl.ac.uk/spm/software/spm12/). Pre-processing was conducted in the following order: slice-timing correction, creation of voxel displacement maps (VDM) using the Field Map toolbox, realignment and unwarping, segmentation of T1 image (unified segmentation algorithm), co-registration of functional images to bias corrected T1, and normalization to MNI space (spatial resolution of 3 × 3 × 3 mm). Spatial smoothing was performed using a Gaussian blurring kernel with 8 mm full-width half-maximum (FWHM). The event onset times were set to the moment when the proposer in the video uttered the final word, thus revealing the fairness/unfairness of the offer (see also Figure 1). The event onset times were convolved with the canonical hemodynamic response function and inserted into a general linear model (GLM). The duration of the event was set to zero. The GLM consisted of four regressors per session: unfair proposal, fair proposal, neutral condition, and reaction time (see also Gospic et al., 2011). Furthermore, six motion parameters were included in the model, in order to correct for residual movement-related variance. High pass filter (cut off frequency = 128 s) was applied. The main contrast for all analyses was unfair > fair offers (this contrast included all offers irrespectively of the subsequent acceptance or rejection by the responder).

Second-level analysis was conducted on the whole-brain. Furthermore, we created a separate mask for the amygdala (dilated by 1 voxel) using wfu_pickatlas, which was used for specific contrasts (Etkin et al., 2004). In order to investigate a potential effect of group and of gender, as well as a potential interaction a 2 × 2 factorial model was created. The main focus was on testosterone _unfair>fair_ > placebo _unfair>fair_ and females _unfair>fair_ > males _unfair>fair_ contrasts. The voxel-wise statistical threshold for all analyses was determined to be *p* < 0.005, uncorrected (extended threshold = 10 voxels) unless otherwise specified. This threshold has been shown to be a desirable balance between Type I and Type II errors (Lieberman and Cunningham, 2009).

## Results

### Hormonal concentration

Due to laboratory issues testosterone levels were lost for five female participants (one for baseline values, and four for values after treatment). The two groups did not differ on their baseline testosterone levels (mean placebo group = 10.3 nmol/L, SEM = 1.6; mean testosterone group = 11.3 nmol/L, SEM = 1.8). Administration of a single dose of testosterone led to a significant increase in testosterone levels compared to administration of placebo gel (*Z* = −5.775, *p* < 0.001). Testosterone levels in men displayed an increase, with mean value just above the normal range (baseline mean = 16.03 nmol/L, SEM = 1; treatment mean = 35.7 nmol/L, SEM = 5.2); testosterone levels in women showed a supra-natural increase (baseline mean = 0.77 nmol/L, SEM = 0.08; treatment mean = 45.6 nmol/L, SEM = 11.8).

### Behavioral

**Figure 2:**
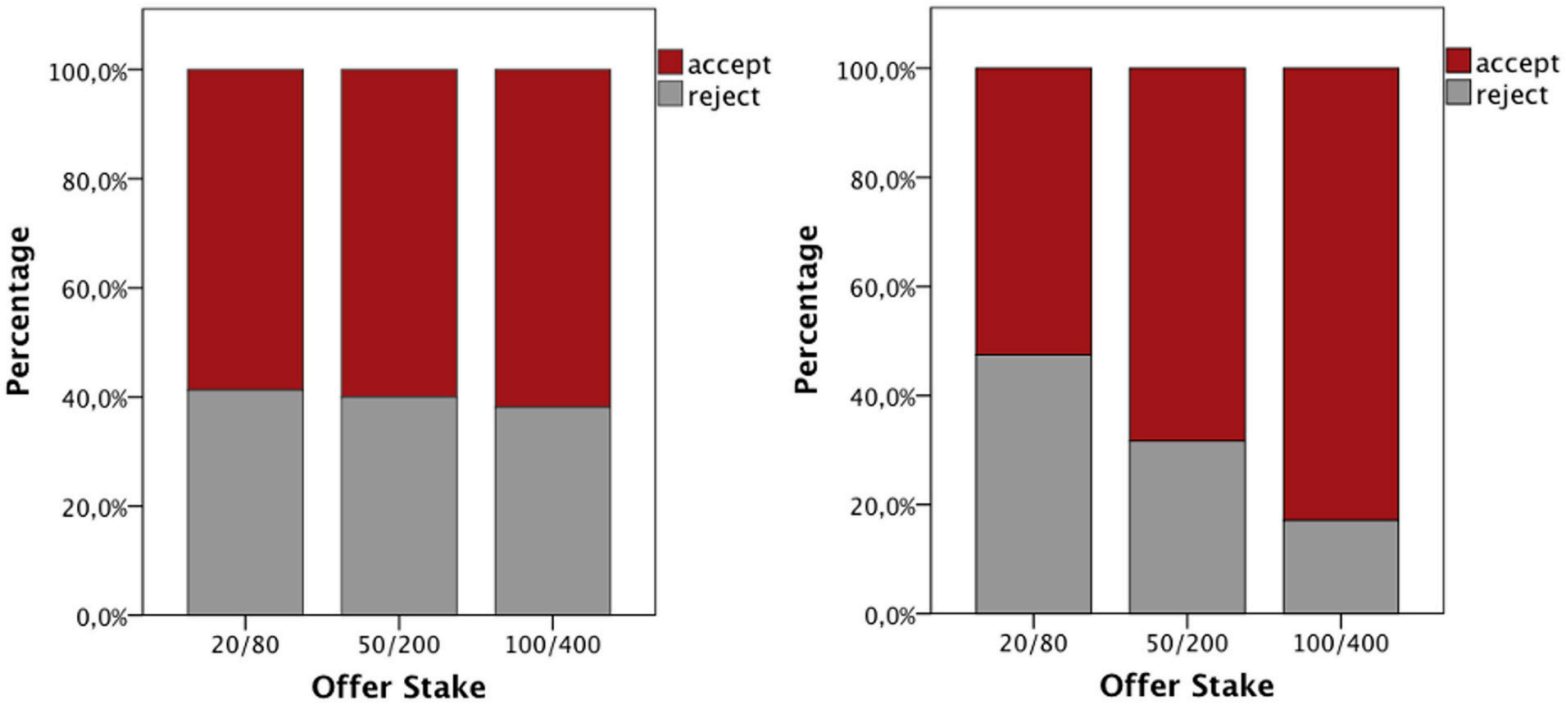
**Women (left) rejected more unfair offers, when the stakes were high, compared to men (right; Wald χ^**2**^ = 5.184, ***p*** = 0.02)**.

As expected, in total participants accepted fair offers in 97.6% of all cases and unfair offers in 62.2% of the cases and there was a significant difference in acceptance rate (χ^2^ = 398.7; *p* < 0.001). The focus was, thus, on the unfair offers. For each participant the rejection ratio was calculated. Mann–Whitney test revealed a statistical trend; the testosterone group accepted more unfair offers than the placebo group (*Z* = −1.483, *p* = 0.138, *r* = −0.18). A GEE probit model was also conducted. Individual responses to unfair offers were treated as the dependent variable; group, gender, and offer stake (20/80, 50/200, and 100/400) were added as the predictors. A significant main effect of offer stake was shown (Wald χ^2^ = 12.827, *p* = 0.002), as participants rejected less the high stake unfair offers. There was a significant gender × offer stake interaction (Wald χ^2^ = 9.129, *p* = 0.01). Subsequent analysis (GEE probit model for individual stake) revealed that women rejected more unfair offers, when the stake was high (100/400; Wald χ^2^ = 5.184, *p* = 0.02; Figure 2). There was no correlation between participants' testosterone levels at baseline or after treatment and rejection rate (*r*_*s*_ = −0.126, *p* = 0.32; *r*_*s*_ = −0.162, *p* = 0.2, respectively).

### No effect of testosterone on ratings of unfairness or likeability

The testosterone treatment did not have an effect on the likeability of the proposers [*t*_(63.730)_ = −1.11, *p* = 0.27, *r* = 0.14] or on the general perception of unfairness (*Z* = −0.11, *p* = 0.91, *r* = −0.01).

### Testosterone effect on psychological traits

**Figure 3:**
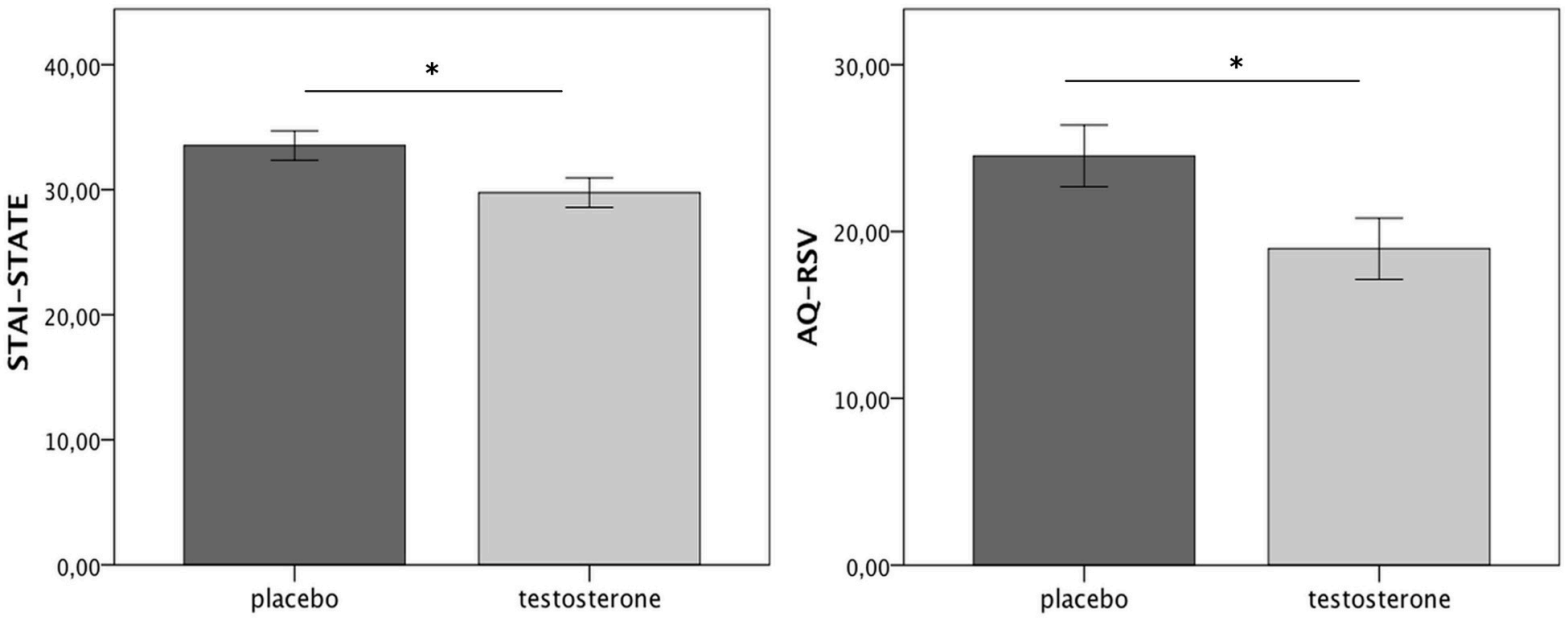
**Testosterone group reported reduced anxiety (left) and aggression (right) after treatment (no significant differences were reported in baseline STAI-STATE or AQ-RSV scores, see Supplementary Material)**. Errors bar refer to S.E. ±1. ^*^p < 0.05.

The testosterone group scored lower on STAI-STATE compared to the placebo (*Z* = −2.15, *p* = 0.03, *r* = −0.26), denoting reduced anxiety after treatment (Figure 3 left). Furthermore, the level of aggression as indicated by the AQ-RSV decreased with the administration of testosterone. Specifically, the participants, who received testosterone, reported lower aggression than the participants, who received placebo (*Z* = −2.264, *p* = 0.024, *r* = −0.27; Figure 3 right). There was no effect of gender on the level of aggression reported after treatment (*Z* = −0.742, *p* = −0.458, *r* = −0.09). A within-group comparison (non-parametric Wilcoxon Signed test) further showed that while there was no difference in the placebo group between the two experimental sessions in terms of AQ-RSV scores (total: *Z* = −0.23, *p* = 0.82, *r* = −0.03; direct: *Z* = −0.3, *p* = 0.76, *r* = −0.04; indirect: *Z* = −0.03, *p* = 0.97, *r* = −0.003), there was a significant decrease in the direct aggression subscale in the testosterone group (*Z* = −2.42, *p* = 0.015, *r* = −0.29). Furthermore, across groups, participants' testosterone levels after treatment correlated negatively with both STATE scores (*r*_*s*_ = −0.244, *p* = 0.052) and total AQ-RSV scores (*r*_*s*_ = −0.261, *p* = 0.038). Finally, participants were not aware of the experimental group they had been assigned to (χ^2^ = 0.611, *p* = 0.434; odds ratio = 1.5).

### fMRI data

All fMRI analysis was performed on the whole brain. Report of uncorrected results implies that they did not survive multiple corrections. Uncorrected results refer to peak-level analysis, unless otherwise specified. The main contrast unfair > fair offers in the whole sample (*N* = 68) yielded higher activation in brain regions that have previously been associated with unfair offers (Sanfey et al., 2003; Gospic et al., 2011; Feng et al., 2014), such as left frontal superior medial, bilateral anterior cingulate, bilateral middle frontal, bilateral insula, left precuneus, right inferior frontal triangularis, bilateral caudate, and left thalamus (*p* < 0.05, FWE; Table 1).

**Table 1:** **Results of unfair > fair offers contrast (***N*** = 68, whole brain analysis, ***p*** < 0.05 FWE, cluster level)**.

**Brain regions**	**MNI coordinates**	**BA**	**Cluster size**	***T***	***Z***
Superior medial left	0, 23, 44	32	826	8.96	7.19
Anterior Cingulum left	−9, 29, 26	32	826	8.77	7.08
Anterior Cingulum right	9, 29, 35	32	826	8.73	7.06
Middle frontal left	−24, 44, 32	46	44	6.12	5.41
Middle frontal right	24, 50, 29	46	71	5.79	5.17
Insula right (anterior insula)	33, 23, −1	47	66	5.61	5.04
Precuneus right	−6, −70, 38	7	13	5.53	4.98
Inferior frontal triangularis right	42, 32, 29	45	26	5.45	4.92
Inferior orbitofrontal left	−30, 23, −4	47	33	5.40	4.88
Caudate left	−9, 8, 5	N/A	6	5.23	4.75
Thalamus left	−9, −4, 2	N/A	2	5.09	4.65
Caudate right	12, 14, 5	N/A	5	5.04	4.60
Insula left (anterior insula)	−42, 17, 5	48	2	4.91	4.51

#### Effect of testosterone on brain activity related to unfair offers

**Figure 4:**
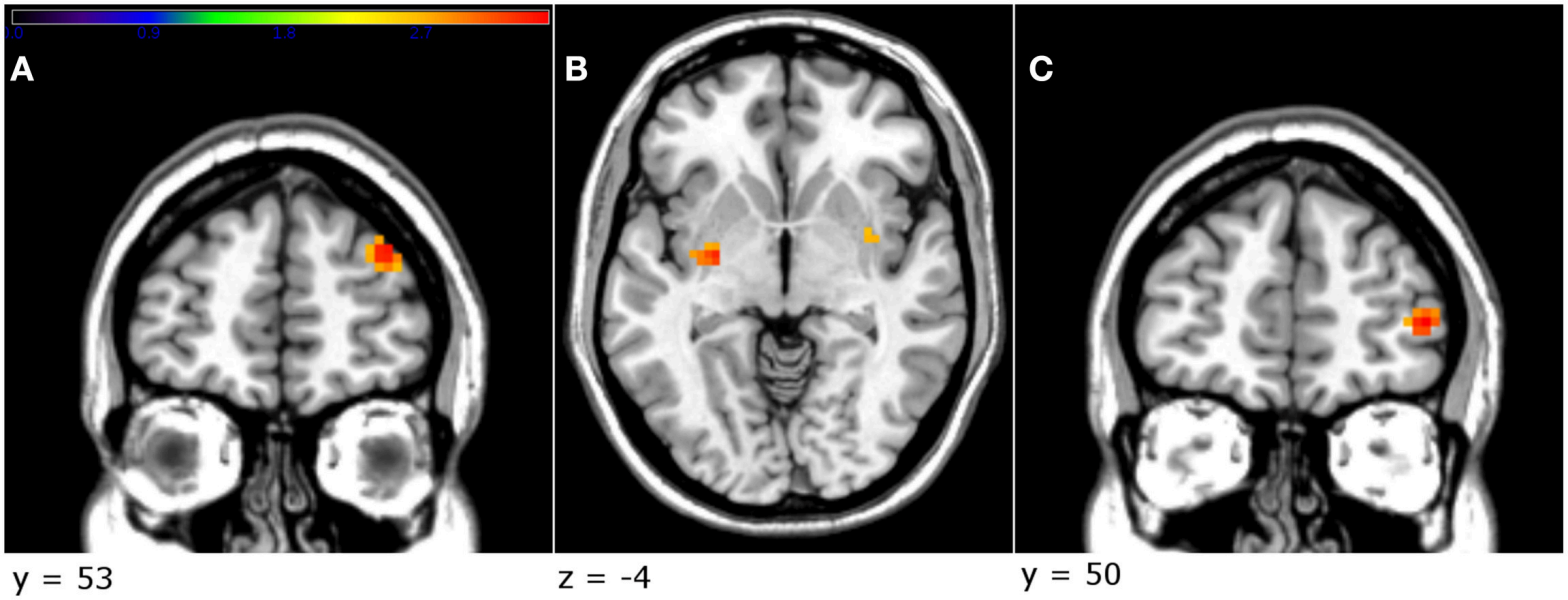
**The testosterone group showed a greater activation in the right dlPFC compared to the placebo group (A)**. In addition, in the testosterone group, a greater decrease in the direct aggression subscale, between sessions, was related to greater activation in the left globus pallidus **(B)** and the right vlPFC **(C)** (*p* < 0.005, uncorrected, peak-level). Threshold bar refers to *T*-values (*T* >2.7).

The factorial design analysis showed an effect of group. An effect of treatment was present in the right dlPFC [*Z* = 3.36, *p* < 0.001, uncorrected, cluster size = 21, MNI coordinates (x, y, z): 33, 56, 29], where the testosterone group displayed greater activation than the placebo (Figure 4A). Furthermore, in order to investigate whether testosterone administration had an effect on the contrast unfair > fair offers in the amygdala, an independent *t*-test was implemented (2nd level analysis). This ROI analysis did not yield any significant results.

#### Effect of testosterone levels on brain activity related to unfair offers

To further investigate the role of testosterone in brain activity, we calculated the difference in testosterone levels between baseline and treatment (TestDiff). Since this difference was close to zero for the placebo group, we focused our analysis only on the testosterone group. TestDiff was then used as a covariate in the contrast unfair>fair offers (regression analysis). This analysis showed a significant positive effect of TestDiff in the left caudate [*Z* = 3.55, *p* < 0.001, uncorrected, cluster size = 27, MNI coordinates (x, y, z): −6, 5, 20] and part of corpus callosum [*Z* = 3.45, *p* < 0.001, uncorrected, cluster size = 28, MNI coordinates (x, y, z): 9, 17, 20]. An additional analysis on the negative effect of TestDiff revealed activation mainly in bilateral temporal regions (see Supplementary Table 4 for more details).

#### Effect of aggression on brain activity related to unfair offers

Due to the fact that there was a difference between the two groups in aggression-related scores, as well as a specific reduction in the direct aggression in the testosterone group, we sought to investigate a potential correlation between direct aggression and brain activity. In order to achieve that, we used as a covariate in the contrast unfair > fair offers for the testosterone group the difference in scoring between the two sessions (dirAggDiff = direct aggression scoring treatment–direct aggression scoring baseline). This analysis yielded a significant negative effect of dirAggDiff in the right ventrolateral prefrontal cortex [vlPFC; *Z* = 3.41, *p* < 0.001, uncorrected, cluster size = 29, MNI coordinates (x, y, z): 45, 50, 5], the left globus pallidus [*Z* = 3.26, *p* = 0.001, uncorrected, cluster size = 16, MNI coordinates (x, y, z): −27, −10, −4], the right putamen [*Z* = 3.13, *p* = 0.001, uncorrected, cluster size = 12, MNI coordinates (x, y, z): 36, −1, −7] and left superior temporal [*Z* = 3.12, *p* = 0.001, uncorrected, cluster size = 21, MNI coordinates (x, y, z): −48, 2, −10; Figures 4B,C].

#### Effect of gender on brain activity related to unfair offers

**Figure 5:**
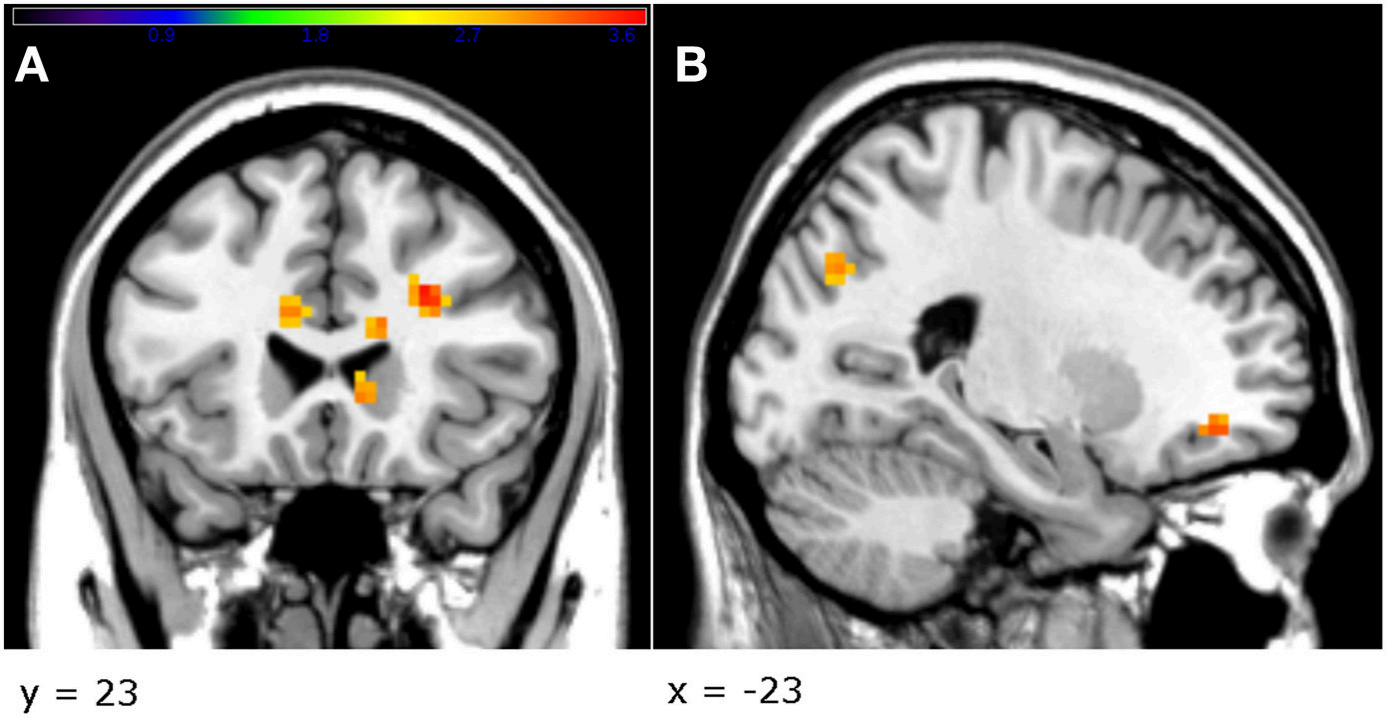
**Female participants activated to a greater extend the striatum and the cingulate (A), as well as the right OFC (B), when viewing unfair offers, in comparison with male participants (***p*** < 0.005, uncorrected, peak-level)**. Threshold bar refers to *T*-values (*T* > 2.7).

The factorial model revealed an effect of gender on brain activity related to unfair offers. Specifically, women displayed greater activation than men in a number of areas, including the right anterior cingulate cortex [cluster size = 91; *Z* = 3.53, *p* < 0.001, uncorrected, MNI coordinates (x, y. z): 27, 26, 26, and *Z* = 3.29, *p* = 0.001, uncorrected, MNI coordinates (x, y, z): 15, 35, 29] and the left superior orbitofrontal cortex [*Z* = 3.37, *p* < 0.001, uncorrected, cluster size = 31, MNI coordinates (x, y, z): −21, 41, −10; Figure 5; see also Table 2]. Furthermore, the factorial analysis did not reveal any significant gender x group interaction.

**Table 2:** **Results of women _**unfair > fair**_ > men _**unfair > fair**_ contrast (***N*** = 68, whole brain analysis, ***p*** < 0.005, uncorrected, extended threshold = 10 voxels)**.

**Brain regions**	**MNI coordinates**	**BA**	**Cluster size**	***T***	***Z***
Anterior cingulate right	27, 26, 26	48	91	3.72	3.53
	15, 35, 29	32	91	3.44	3.29
Superior orbitofrontal left	−21, 41, −10	11	31	3.54	3.37
	−21, 32, −7	47	31	2.78	2.69
N/A	15, 23, 20	N/A	10	3.14	3.02
Cingulate region left	−12, 32, 8	11	41	3.13	3.01
	−9, 23, 23	N/A	41	3.09	2.97
Caudate right	9, 23, −1	23	31	3.11	2.99
	9, 29, 11	25	31	2.83	2.98
Precuneus left	−6, −76, 53	7	14	3.10	3.66
Middle orbitofrontal right	33, 44, −4	47	11	3.07	2.96
Superior occipital left	−24, −70, 35	19	19	3.07	2.96

#### Role of the amygdala in rejection of unfair offers

In an attempt to further investigate a potential role of the amygdala in the Ultimatum Game, the unfair offers that were rejected were contrasted with the unfair offers that were accepted (based on Gospic et al., 2011). This contrast was conducted only for the participants, who had accepted and rejected more than one unfair offer in both fMRI sessions (*N* = 12). This analysis did not reveal any significant differences in amygdala activation.

## Discussion

In the present study a single dose of exogenous testosterone was applied to male and female participants prior to engaging in a standard Ultimatum Game inside an MRI scanner. This is the first fMRI study that investigated the role of testosterone in both genders in the same experiment.

An important finding of the present study is that we successfully manipulated testosterone levels in both genders using the same dose. Sixty milligram of testosterone led to significantly elevated testosterone levels in men, albeit remaining just above the normal range. In women, the same dose of exogenous testosterone led to well above normal range of testosterone. It is of note that the presently reported range of hormonal values was similar to the one reported by previous studies, using different methodology (Tuiten et al., 2000; Eisenegger et al., 2013). Importantly, the subjects were unaware of which experimental group they belonged to, as reported in the post-experimental questionnaire. It should also be mentioned that the present methodology did not allow for verification of the application procedure. The application of the gel in the presence of the experimenter is thus recommended for future studies.

### Testosterone and the ultimatum game

The present study robustly reproduced findings from previous experimental studies of the Ultimatum Game including lower acceptance of unfair proposals and activational findings with increased activity areas such as dlPFC and insula. In terms of behavior in the Ultimatum Game, the administered dose did not induce significant changes in the responders' responses. Nevertheless, the participants who received testosterone tended to accept a greater number of unfair offers compared to the participants who received placebo gel. Furthermore, they reported significantly lower levels of negative mood (as indexed by anxiety and aggression scores), which corroborates with the aforementioned behavioral trend (Pillutla and Murnighan, 1996; Harle and Sanfey, 2007). Reduced levels of anxiety after testosterone administration are supported by previous studies (Eisenegger et al., 2011) suggesting an anxiolytic role of testosterone. The relation between testosterone and aggression, though, is a more complex one. Our present finding that testosterone administration was related to lower aggression scores was unexpected. Nevertheless, there are a few possible reasons accounting for that. To start with, in the present study aggression was measured by a self-report questionnaire, which might not have been the most reliable measure of aggression. Previous studies using the AQ questionnaire have shown inconclusive findings with regards to increased testosterone levels and aggression scores, i.e., a null effect (Pope et al., 2000; O'Connor et al., 2002) or a positive relation (Kouri et al., 1995). More importantly, recent evidence points toward a complex relation between testosterone and aggression in humans (Carre and Olmstead, 2015). Briefly, studies have shown that the relation between aggression and testosterone is probably mediated by other factors, such as dominance, impulsivity, and context (Archer, 2006; Carre and Olmstead, 2015). Especially, with regards to the latter, endogenous testosterone levels have been shown to fluctuate depending on environment (i.e., rising in a competitive environment and in winners) while exogenous testosterone administration has been shown to promote cooperation under specific conditions (Everett et al., 2015). Thus, an increase in testosterone levels *per se* might not be sufficient to lead to an increase in aggression. It is also of note that in our study, the environment might not have been perceived as competitive, but as cooperative, thus restricting or even reversing the effect of testosterone on aggression.

Our behavioral findings are not in line with previous studies, where high levels of exogenous testosterone were either related to a tendency toward increased rejection rate (albeit not significant) (Zak et al., 2009) or did not affect performance (Zethraeus et al., 2009; Eisenegger et al., 2010). The reasons for this discrepancy could be multifaceted, notably the methodological differences between the present and past studies. Our study consisted of a mixed gender sample that received the same testosterone dose. Contrary to the study by Zak et al. (2009), participants engaged in the Ultimatum Game only as responders and were tested ~3.5–4 h after treatment (instead of a 16 h gap). Our experimental design was closer to the one implemented by Eisenegger et al. (2010). However, our study differed on the specifics of the Ultimatum Game and on the fact that the female participants in our study were using contraceptive pills, which could have been one of the factors accounting for the behavioral differences observed.

The present results suggest that testosterone administration did not increase the emotional response toward unfair offers and possibly even created a bias toward the deliberation system. This is supported by the fMRI findings, according to which the participants who received testosterone exhibited a greater activation (at significance threshold level) of the right dlPFC, when viewing unfair offers, implying that they recruited to a greater extent cortical areas related to reappraisal and cognitive control (Grecucci et al., 2013; Haruno et al., 2014). Furthermore, in the testosterone group, an increase of testosterone levels was associated with increased activation in the left dorsal caudate, an area that is implicated in choice value and goal-directed behavior (Grahn et al., 2008; Wunderlich et al., 2012) and is connected to the dlPFC (Lehéricy et al., 2004). The inverse correlation of testosterone levels observed mainly in temporal areas could be related to empathy and mentalizing (Schnell et al., 2011). Two things are also of note; in the testosterone group a decreased score in direct aggression was related to a greater activation in the left globus pallidus and right vlPFC, areas that have been implicated in goal-directed behavior and acceptance of unfair offers respectively (Tabibnia et al., 2008; Arimura et al., 2013). Finally, the testosterone group in our study exhibited a greater activation in the striatum in a passive monetary task, when receiving rewards (see Supplementary Material), potentially denoting a bias toward less aggressive behavior in the Ultimatum Game. Notwithstanding, a caveat is that all activations were below significance level after correction, thus they must be interpreted with caution.

The suggested role of testosterone in biasing behavior toward controlled processes is intriguing. Previous human and animal studies have shown that testosterone could lower discounting and promote delayed rewards (Takahashi et al., 2008; Wood et al., 2013). In the context of the Ultimatum Game, acceptance of unfair offers could be perceived as a delayed reward, as responders have to wait until the end of the experiment to receive the money (rejection is associated with immediate reward, as the responder punishes financially the proposer; Crockett et al., 2010). Another line of research has also shown that testosterone does not always promote aggression, but could also lead to cooperation and pro-social behavior, depending on the context. Indeed, Diekhof et al. (2014) showed that in the Ultimatum Game participants accepted more unfair offers by proposers that belong to their group (in-group) compared to proposers that were out-group members. Furthermore, this behavior was positively correlated with endogenous testosterone levels (in an intergroup competition context). The suggested role of testosterone in cooperation has also been observed in other economic games, such as the Prisoner's Dilemma (Reimers and Diekhof, 2015), and the trust game (Boksem et al., 2013).

It is thus possible that our participants, who received testosterone, tended to control the immediate emotional reaction to punish the proposer, and to wait instead for the delayed monetary reward. This behavioral tendency could have also been influenced by a bias toward exhibiting pro-social behavior (i.e., they might have perceived the proposers as in-group members). Notwithstanding, caution should be exercised when interpreting the present findings, due to the limitations of the present study (i.e., no direct testing of pro-social behavior or of delay discounting, plus uncorrected fMRI data), as well as due to the inconclusive role of the dlPFC in the Ultimatum Game (Sanfey et al., 2003, 2014; Knoch et al., 2006; Baumgartner et al., 2011; Ruff et al., 2013).

### Role of amygdala in the ultimatum game

In the present study we did not replicate the results by Gospic et al. (2011), according to which greater amygdala activation was associated with rejected unfair offers compared to accepted unfair offers. The reasons for this discrepancy are not known. It should be mentioned though that weak left amygdala activation was present, when the threshold was lowered to *p* < 0.05, uncorrected [*Z* = 1.99, *p* = 0.023 uncorrected, cluster size = 12, MNI coordinates (x, y, z): −27, −7, −13]. Amygdala activation could be seen as an immediate reactive response to unfairness, leading participants to forfeit their self-interest, and punish the unfair proposer (Gospic et al., 2011). Nevertheless, caution should be exercised, as the present findings do not allow us to further extrapolate on the role of amygdala in the Ultimatum Game.

### Gender and the ultimatum game

In the present study, there was no main effect of gender in terms of responses in the Ultimatum Game. However, there was a significant interaction between gender and offer stake, wherein female responders were more likely to reject unfair offers when the monetary stakes were high, compared to male responders. Thereby, women were willing to forfeit more money to uphold fairness than men. This finding is in line with some previous studies, which showed that men have a lower minimum acceptance offer than women (McGee and Constantinides, 2013) and that women are driven more by reciprocity and fairness (Croson and Gneezy, 2009).

With regards to the fMRI analysis, women tended to display greater activation in areas including the anterior cingulate, the caudate nucleus and the orbitofrontal cortex, suggesting a gender difference. This pattern of activation could be seen as a manifestation of a greater activation of the emotional system (Sanfey et al., 2006), which in turn could imply a higher rejection rate. The anterior cingulate cortex has been shown to be activated in relation to unfair offers (Sanfey et al., 2003) and to be associated with higher expectation in social decision-making (Chang and Sanfey, 2013), while activation in the striatum could be related to the expected satisfaction derived from altruistic punishment (De Quervain et al., 2004). The greater activation of the OFC is interesting. Lateral OFC is involved in reward-related processes and in evaluation of negative reinforcers, which could lead to a change in behavior (Kringelbach, 2005). Thus, it is possible that when faced with an unfair offer, women adapted their behavior accordingly by choosing to reject that offer, a decision potentially driven by their higher degree of inequity aversion and fairness preference compared to men.

Finally, it could be possible that the aforementioned behavioral gender difference is related to the behavioral profile of the male participants that we observed in our previous study on hypothetical bias (Gospic et al., 2014). Despite the differences between the two experimental paradigms and settings, it might be plausible that in both scenarios, men, when faced with real decisions, opted to behave in a more generous and cooperative manner than women, by accepting the proposal that was being offered to them. The reason behind this behavior is not known. It could be speculated that in both studies an implicit effect of the experimenter's gender was present (female experimenter). Indeed prior evidence exists indicating that participants behave more altruistically and reciprocate more in a trust game, when the experimenter is a woman (Innocenti and Pazienza, 2006). Furthermore, studies on pain have shown that men report less pain and negative emotions in front of a female experimenter than in front of a male experimenter (Aslaksen et al., 2007). However, as the current study was not designed to investigate a potential experimenter effect, this speculation should be treated with caution. An interesting future study would thus be to manipulate the gender of the experimenter in an Ultimatum Game setting.

## Conclusions

The role of testosterone in social interaction and economic behavior has been the focus of extensive research. The present study aimed to further investigate the potential effect of testosterone on the Ultimatum Game in a mixed sample of healthy volunteers. The results of the present study, albeit being weak, suggest that exogenous testosterone administration decreases anxiety and direct aggression, and creates a bias toward higher acceptance of unfair offers - an effect that could be mediated by greater activation in the dlPFC and the caudate. Higher acceptance of unfair offers could be related to control processes and greater activation of the deliberation system. Furthermore, our study shows possible differences between men and women in rejecting higher stakes–a difference that has not been tested previously. Given the inconsistency of past literature and the limitations of the present study, future studies are needed in order to replicate and expand upon the above findings.

## Author contributions

EK acquired, analyzed and interpreted data, and drafted and revised the work; JB provided substantial contribution to the design of the work, and revised the work; PP provided substantial contribution to the design of the work, and revised the work; MI provided substantial contribution to the design of the work, and revised the work. All authors provided their final approval of the submitted version and agreed to be accountable for all aspects of the work.

## Funding

The present study was supported by a Vetenskapsrådet (VR) grant (20121999) granted to MI.

### Conflict of interest statement

The authors declare that the research was conducted in the absence of any commercial or financial relationships that could be construed as a potential conflict of interest.
