# Supplementary material for: Testosterone Administration Related Differences in Brain Activation during the Ultimatum Game
Source: Front Neurosci. 2016 Mar 1;10:66. doi: 10.3389/fnins.2016.00066 (PMC4771731; doi:10.3389/fnins.2016.00066)
Supplement: Supplementary file 1 [file DataSheet1.DOCX]

Supplementary Material

**Testosterone administration related differences in brain activation during the Ultimatum Game**

Eleni Kopsida, Jonathan Berrebi, Predrag Petrovic, Martin Ingvar*

*** Correspondence:** Martin Ingvar: martin.ingvar@ki.se

# Supplementary Methods

## Personality traits

During the first session, in order to ascertain that there were no baseline differences between the two experimental groups, all participants completed the following questionnaires:

1. Kinsey scale ([Kinsey *et al.*, 1948](#_ENREF_3))
2. Rosenberg Self-Esteem scale ([Rosenberg *et al.*, 1995](#_ENREF_8))
3. BDI (Beck Depression Inventory) ([Beck *et al.*, 1988](#_ENREF_1))
4. STAI-STATE Anxiety Questionnaire ([Forsberg and Björvell, 1993](#_ENREF_2))
5. STAI-TRAIT Anxiety Questionnaire ([Forsberg and Björvell, 1993](#_ENREF_2))
6. AQ-RSV (Aggression Questionnaire – Revised Swedish Version) ([Prochazka and Ågren, 2001](#_ENREF_7))
7. TriPM (Triarchic Psychopathy Measure) ([Patrick *et al.*, 2009](#_ENREF_5))
8. PDI (Peters et al. Delusions Inventory) ([Peters *et al.*, 2004](#_ENREF_6))

## Wheel of Fortune (WoF)

Prior to the Ultimatum Game, participants engaged in a passive computerized lottery task. Participants were given an endowment of 50 SEK and were told that during the task, they had the chance to win or lose money, or break even. They were explicitly told that the outcome of each trial was based on luck and that they could not influence it in any way. They were also informed that part of their financial compensation would be the total amount of money accumulated during the task.

The task was comprised of 72 trials, split into two sessions. At the beginning of each trial a wheel was presented, divided into three parts: “won”, “lost”, “try again” (original wording was in Swedish; see Supplementary Figure 1). Participants were asked to press a button in order to spin the wheel. The wheel span for 2-3 secs and was followed by a fixation cross (jittered between 3.5 and 6 secs). After the fixation period, an arrow pointed towards the lottery outcome, accompanied by the monetary amount (Note: if arrow pointed to “try again”, no monetary amount was attached to it) (2 secs). Trials were separated by a fixation period, lasting between 3.5 and 6 secs. At the end of each session, participants received feedback on the total amount of money that they currently had in their account.

## Data acquisition and analysis

Data acquisition and analysis followed the same protocol as in the Ultimatum Game, described in the main text. The WoF was split into two fMRI sessions (36 trials each). Regarding data analysis, the GLM consisted of six regressors per session: click_positive (pressing of button associated with subsequent “win”), click_negative (pressing of button associated with subsequent “loss”), click_neutral (pressing of button associated with subsequent “try again”), outcome_positive (“win”), outcome_negative (“loss”), outcome_neutral (“try again”). In addition, six motion parameters were included in the model, in order to correct for residual movement-related variance. High pass filter (cut off frequency = 128s) was applied. For the second-level analysis, a striatum ROI was created using wfu_pickatlas (Maldjian et al., 2003). The analysis focused on the win>loss contrast. Due to technical issues, there was no WoF data for two participants (N=66).

# Supplementary results

## Statistical analysis of personality traits

Non-parametric Mann-Whitney test was applied for the analysis of the above questionnaires (Supplementary Table 1). Bonferroni correction for multiple comparisons was used (0.05/8 = 0.00625).

## Correlation of rejection rate and personality traits

A correlation analysis was conducted between rejection rate and the following personality traits: Rosenberg, BDI, STAI-TRAIT, TRiPM and PDI. There was no significant correlation between rejection rate and any of these measures (Supplementary Table 2). There was, though, a correlation between the TriPM subscale of meanness and rejection rate (*r*_s_ = .303, p = .012).

## Wheel of Fortune (WoF) analysis

The main contrast win>loss in the whole sample (N = 66; ROI analysis; voxel-wise threshold: p < .05 FWE) yielded higher activation in right caudate (Z = 3.76, p = .025, FWE corrected, cluster-level, MNI coordinates (x, y, z): 18, -13, 20) and left putamen (Z = 3.61, p = .034, FWE corrected, cluster-level, MNI coordinates (x, y, z): -21, 11, -7; Z = 3.55, p = .034, FWE corrected, cluster-level, MNI coordinates (x, y, z): -30, -16, 2) (Supplementary Figure 2). Group comparison analysis between testosterone and placebo for the same contrast (ROI analysis; voxel-wise threshold: p < .005, uncorrected) revealed a higher activation in the left putamen for the testosterone group (Z = 3.06, p = .001, uncorrected, MNI coordinates (x, y, z): -27, -16, 11) (Supplementary Figure 3).

# Supplementary Figures and Tables

## Supplementary Figures


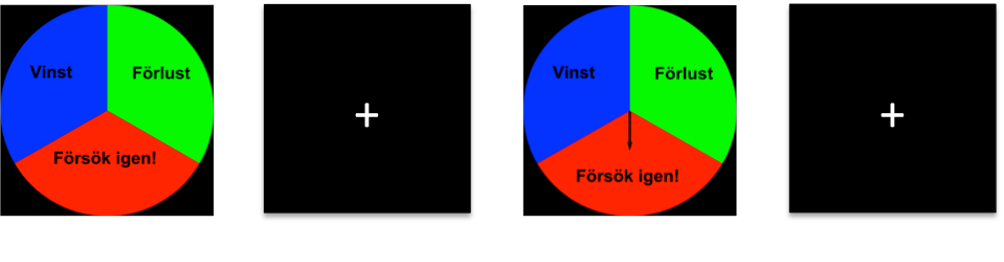


**Supplementary Figure 1.** Example of an experimental trial of the WoF. Participants were instructed to press a button, in order to initiate spinning (A.). Fixation periods (B. and D.) were jittered (3.5 to 6 secs). The outcome was shown for 2 secs (C.).

**
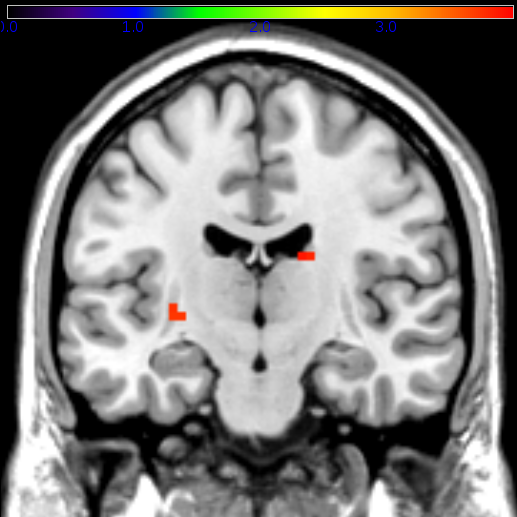
**

**Supplementary Figure 2.** Across participants, there was a greater activation of the striatum when the outcome of the trial was positive (win) than when it was negative (loss) (p < .05, FWE corrected; y = -15). Threshold bar refers to T values (T = 3.64).


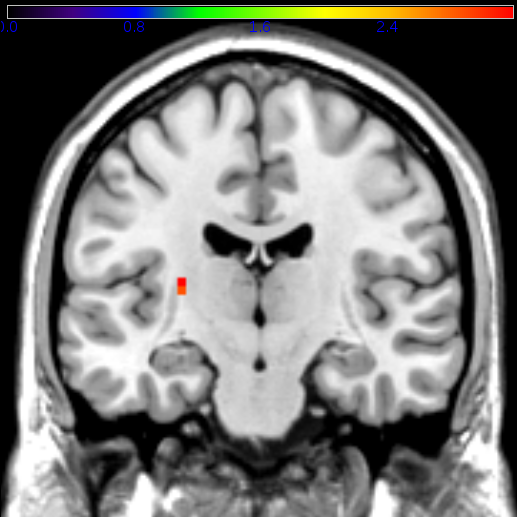


**Supplementary Figure 3.** Testosterone group exhibited a greater activation in the left putamen, associated with the win>loss contrast, compared to placebo group (p = .001, uncorrected; y = -16). Threshold bar refers to T values (T = 2.65).

## Supplementary Tables

**Supplementary Table 1**. Testosterone and placebo group did not significantly differ in their baseline personality traits, as measured during the first lab visit. The significant difference on the STAI-TRAIT did not survive multiple comparisons.

| **Questionnaire** | **Z** | **P value** |
| --- | --- | --- |
| Kinsey | - .655 | .512 |
| Rosenberg scale | -1.112 | .266 |
| BDI | -1.446 | .148 |
| STAI-TRAIT | -2.629 | .009 |
| STAI-STATE | -1.253 | .210 |
| AQ-RSV  Direct aggression  Indirect aggression  Total aggression | -.824  -1.996  -1.952 | .410  .046  .051 |
| TRiPM | -1.798 | .072 |
| PDI | -1.998 | .046 |

**Supplementary Table 2**. There was no significant correlation between personality traits and rejection of unfair offers in the Ultimatum Game (Bonferroni correction; 0.05/5=0.01).

| **Questionnaire** | ***r*_s_** | **P value** |
| --- | --- | --- |
| Rosenberg scale | -.190 | .121 |
| BDI | .219 | .073 |
| STAI-TRAIT | .164 | .180 |
| TRiPM | .188 | .124 |
| PDI | .026 | .835 |

**Supplementary Table 3.** Whole brain analysis (p < .001, uncorrected, peak – level, cluster threshold = 0) for the contrast unfair > fair offers (N=68).

| **Brain regions** | **MNI coordinates** | **BA** | **Cluster size** | **T** | **Z** |
| --- | --- | --- | --- | --- | --- |
| Superior medial left  Anterior Cingulum left  Anterior Cingulum right | 0, 23, 44  -9, 29, 26  9, 29, 35 | 32  32  32 | 2769  2769  2769 | 8.96  8.77  8.73 | 7.19  7.08  7.06 |
| Insula right | 33, 23, -1 | 47 | 279 | 5.61 | 5.04 |
| Precuneus left | -6, -70, 38 | 7 | 119 | 5.53 | 4.98 |
| Insula left  Caudate left  Thalamus left | -30, 23, -4  -9, 8, 5  -9, -4, 2 | 47  N/A  N/A | 729  729  729 | 5.40  5.23  5.23  5.09 | 4.88  4.75  4.75  4.65 |
| Brainstem/PaG | 3, -28, -22 | N/A | 18 | 4.64 | 4.29 |
| Angular right | 42, -49, 35 | 40 | 35 | 4.01 | 3.77 |
| Inferior parietal left | -45, -55, 41 | 39 | 34 | 3.82 | 3.61 |
| Angular left | -27, -49, 32 | 40 | 4 | 3.39 | 3.24 |
| Superior Orbitofrontal right | 27, 56, -4 | 11 | 6 | 3.37 | 3.22 |
| Brainstem | -3, -25, -7 | N/A | 1 | 3.37 | 3.22 |
| Mid frontal left | -39, 8, 38 | 44 | 2 | 3.28 | 3.14 |
| N/A | -3, -31, 23 | 23 | 2 | 3.26 | 3.12 |
| N/A | 6, -31, 20 | N/A | 1 | 3.22 | 3.09 |

**Supplementary Table 4**. Regions related to a negative effect of TestDiff on unfair > fair offers contrast (p < .005, uncorrected, peak – level, extended threshold = 10 voxels)

| **Brain regions** | **MNI coordinates** | **BA** | **Cluster size** | **T** | **Z** |
| --- | --- | --- | --- | --- | --- |
| Superior temporal left | -60, -7, 8  -63, -13, 14  -54, 8, -1 | 48  22  38 | 137  137  137 | 4.34  4.33  3.43 | 3.71  3.71  3.08 |
| Supramarginal left | -66, -49, 26 | 22 | 38 | 3.95 | 3.45 |
| Insula left | -30, -1, 11 | 48 | 11 | 3.76 | 3.31 |
| Middle temporal right | 66, -46, 14  69, -40, 5 | 22  22 | 34  34 | 3.72  3.31 | 3.29  2.99 |
| Superior temporal right | 63, -7, 8 | 22 | 31 | 3.59 | 3.19 |
| Supplementary motor area right | 3, -7, 65 | 3 | 25 | 3.49 | 3.12 |
| Middle cingulate left | -3, 5, 41  0, -7, 47 | 24  N/A | 18  18 | 3.38  3.08 | 3.04  2.81 |
| Superior temporal left | -63, -34, 17 | 42 | 10 | 3.34 | 3.01 |
| Inferior temporal right | 51, -46, -16  60, -49, -13 | 20  37 | 11  11 | 3.06  2.95 | 2.80  2.70 |
